# Urinary pentosidine level is associated with grip strength and gait speed in community-dwelling adults: a cross-sectional study

**DOI:** 10.1186/s12891-021-04279-5

**Published:** 2021-04-26

**Authors:** Kenta Moriwaki, Hiromi Matsumoto, Chika Tanimura, Mari Osaki, Hideki Nagashima, Hiroshi Hagino

**Affiliations:** 1 Department of Orthopedic Surgery, Faculty of Medicine, Tottori University, Nishicho 36-1, Yonago, Tottori, 683-8504 Japan; 2 Department of Orthopedic Surgery, Misasa Onsen Hospital, Yamada 690Misasa, Tottori, 682-0122 Japan; 3 Department of Rehabilitation, Faculty of Health Science and Technology, Kawasaki University of Medical Welfare, Matsushima 288, Kurashiki, Okayama, 701-0193 Japan; 4 School of Health Science, Faculty of Medicine, Tottori University, Nishicho 86, Yonago, Tottori, 683-8504 Japan; 5 Rehabilitation Division, Tottori University Hospital, Nishicho 36-1, Yonago, Tottori, 683-8504 Japan

**Keywords:** Muscle and bone interactions, Osteoporosis, Sarcopenia, 25-hydroxyvitamin D, Biomarker, Physical function, Skeletal muscle mass index, Insulin-like growth factor-1, Speed of sound, Estimated glomerular filtration rate

## Abstract

**Background:**

Muscle and bone interactions might be associated with osteoporosis and sarcopenia. Urinary pentosidine and serum 25-hydroxyvitamin D (25(OH)D) might affect muscle and bone interactions. It is unclear whether these biomarkers are affected by age and sex or play a role in muscle and physical functions. We aimed to investigate the association between urinary pentosidine and serum 25(OH)D levels with muscle mass, muscle strength, and physical performance in community-dwelling adults.

**Methods:**

Two-hundred and fifty-four middle-aged and elderly adults were enrolled. There was no significant difference in age between 97 men (75.0 ± 8.9 years) and 157 women (73.6 ± 8.1 years). The skeletal muscle mass index (SMI), grip strength, and gait speed were assessed. The urinary pentosidine level was measured. We evaluated the association of urinary pentosidine and serum 25(OH)D levels with age and sex (student’s t-test) and correlations between biomarker and each variable (Pearson’s correlation coefficients). Multiple regression analysis was performed with grip strength and gait speed as dependent variables and with age, height, weight, body mass index (BMI), speed of sound (SOS), SMI, glycated hemoglobin (HbA1c), estimated glomerular filtration rate (eGFR), 25(OH)D, and pentosidine as independent variables using the stepwise method.

**Results:**

The urinary pentosidine level was negatively correlated with grip strength, gait speed, eGFR, and insulin-like growth factor-1 (IGF-1) in men and with SOS, grip strength, and gait speed in women. The serum 25(OH)D level was positively correlated with IGF-1 in women and grip strength in men. Grip strength was associated with age, height, and pentosidine in men and height and pentosidine in women. Gait speed was associated with age, BMI, and pentosidine in men and age, height, and pentosidine in women.

**Conclusion:**

Urinary pentosidine levels are significantly associated with grip strength and gait speed and may serve as a biomarker of muscle and bone interactions.

## Background

In recent years, there has been a considerable focus on the relationship between osteoporosis and sarcopenia. In previous studies, osteoporosis patients with fragility fractures were found to have a high prevalence of sarcopenia [1, 2]. Muscle tissue and bone metabolism interact mechanically, functionally, and with associated endocrine functions [3]. The interactions between muscle and bone might help to describe the relationship between osteoporosis and sarcopenia. We focused on urinary pentosidine and serum 25-hydroxyvitamin D (25(OH)D) levels as biomarkers that might affect muscle and bone interactions.

Pentosidine is a representative cross-linked structure of advanced glycation end products (AGEs), which are induced by the oxidation of bone collagen crosslinks [4]. Pentosidine is a bone collagen and its level in urine increases with age [5, 6]. Urinary pentosidine was reported to be a risk factor for fragile fractures, independent of age, and bone density in postmenopausal women [7]. On this basis, in recent years, urinary pentosidine has been used as a bone quality marker.

Ergocalciferol and cholecalciferol (D3) are incorporated into the diet; vitamin D3 is also synthesized in the skin and undergoes hydroxylation in the liver to become 25-hydroxyvitamin D (25(OH)D). 25(OH)D is stable in the blood, and its level has recently been reported to be useful for assessing vitamin D sufficiency [8]. 25(OH)D is hydroxylated in the kidney to become 1,25(OH)2D, an active vitamin D, which binds to the nuclear receptor and vitamin D receptor and regulates calcium absorption in the intestinal tract [9]. An association of low serum 25(OH)D levels with osteoporotic fractures has been reported [10–12].

We found that insulin-like growth factor-1 (IGF-1) is an important biomarker not only of muscle tissues but also of bones in community-dwelling middle-aged and elderly adults [13]. We similarly focused on urinary pentosidine and serum 25(OH)D levels as biomarkers that might affect muscle and bone interactions. It is unclear whether these biomarkers are affected by age and sex or if these play a causal role in muscle and physical functions. Grip strength and gait speed are diagnostic criteria for sarcopenia and are important factors that determine physical function [14]. This study aimed to investigate the association between urinary pentosidine and serum 25(OH)D levels with muscle mass, muscle strength, and physical performance in community-dwelling adults.

## Methods

### Study setting and participants

This cross-sectional study used data of participants enrolled in the Good Aging and Intervention Against Nursing Care and Activity Decline (GAINA) study in the town of Hino, Tottori Prefecture, Japan [15–18]. A total of 254 middle-aged and elderly adults participated, and there was no significant difference with regard to the age between 97 men (75.0 ± 8.9 years) and 157 women (73.6 ± 8.1 years) in this study in 2016 [13]. Information on the participants’ characteristics was obtained from their medical records. The speed of sound (SOS) in the right calcaneus, skeletal muscle mass index (SMI), grip strength, and gait speed were measured using previously reported methods [13].

### Biomarker assessments

Blood samples were taken before the assessment of body structure and physical function parameters. Serum creatinine and glycated hemoglobin (HbA1c) levels were measured. The estimated glomerular filtration rate (eGFR) was calculated using the following formula:

$$ \mathrm{eGFR}=194\times {\mathrm{serum}\ \mathrm{creatinine}}^{-1.094}\times {\mathrm{age}}^{-0.287}\times 0.739 $$

The serum IGF-1 and parathyroid hormone (PTH) levels were measured using a radioimmunoassay and an electrochemiluminescence immunoassay (ECLIA) kit (Elecsys PTH; Roche Diagnostics GmbH, Mannheim, Germany), respectively. Serum 25(OH)D levels were measured using an ECLIA kit (Elecsys Vitamin D total; Roche Diagnostics GmbH, Mannheim, Germany). The limit of quantification is the lowest analyte level that can be reproducibly measured with an intermediate precision coefficient of variance of ≤20%.

The urinary pentosidine levels were measured using an enzyme-linked immunosorbent assay (ELISA) kit (MARKIT-M urinary pentosidine, SB Bioscience Co., Ltd., Tokyo, Japan). The ELISA kit consisted of polyclonal anti-pentosidine IgG and a secondary antibody, and the accuracy, precision, and reliability of this kit were evaluated. In brief, the limit of blank and the limit of detection were 4.25 and 6.24 pmol/mL, respectively. The intra-assay and inter-assay coefficients of variation were < 5%. The spiking and dilution recoveries were 101.4 and 100.5%, respectively. An analysis of cross-reactivity against seven compounds representative of AGEs and with structures close to pentosidine revealed no significant cross-reactivity. The comparability between the values obtained from high-performance liquid chromatography (HPLC) and ELISA (in the same urine samples) was r = 0.815 [19]. Serum creatinine, HbA1c, eGFR, and IGF-1 levels were measured in August 2016 [13]; serum PTH and 25(OH)D levels were measured in June 2018; urinary pentosidine levels were measured in January 2019.

### Statistical analyses

We assessed the association of urinary pentosidine and serum 25(OH)D levels with age and sexes using the student’s t-test and correlations between biomarker and each variable on Pearson’s correlation coefficients (|r| = 0.00–0.19: very weak, |r| = 0.20–0.39: weak, |r| = 0.40–0.59: moderate, |r| = 0.60–0.79: strong, and |r| = 0.80–1.0: very strong). Finally, multiple regression analysis was performed with grip strength and gait speed as dependent variables and with age, height, weight, body mass index (BMI), SOS, SMI, HbA1c, eGFR, 25(OH)D, and pentosidine as independent variables in men and women using the stepwise method. We judged multicollinearity using a variance inflation factor. All statistical analyses were performed by SPSS statistical software (version 24 for Windows; IBM Co., Tokyo, Japan). A *p*-value of < 0.05 was considered statistically significant.

## Results

There was no significant difference in the mean age between men (75.0 ± 8.9 years) and women (73.6 ± 8.1 years) (*p* = 0.198). BMI was significantly higher in men (23.0 ± 2.5 kg/m^2^) than in women (22.0 ± 3.1 kg/m^2^) (*p* = 0.014). The right calcaneal SOS (1503.6 ± 28.3 vs 1483.4 ± 20.2 m/s), SMI (7.5 ± 0.8 vs 6.1 ± 0.8 kg/m^2^), and grip strength (34.9 ± 7.4 vs 23.0 ± 4.5 kg) were significantly higher in men than in women (*p* < 0.001). Gait speed and IGF-1 were not significantly different between sex [15].

**Fig. 1:**
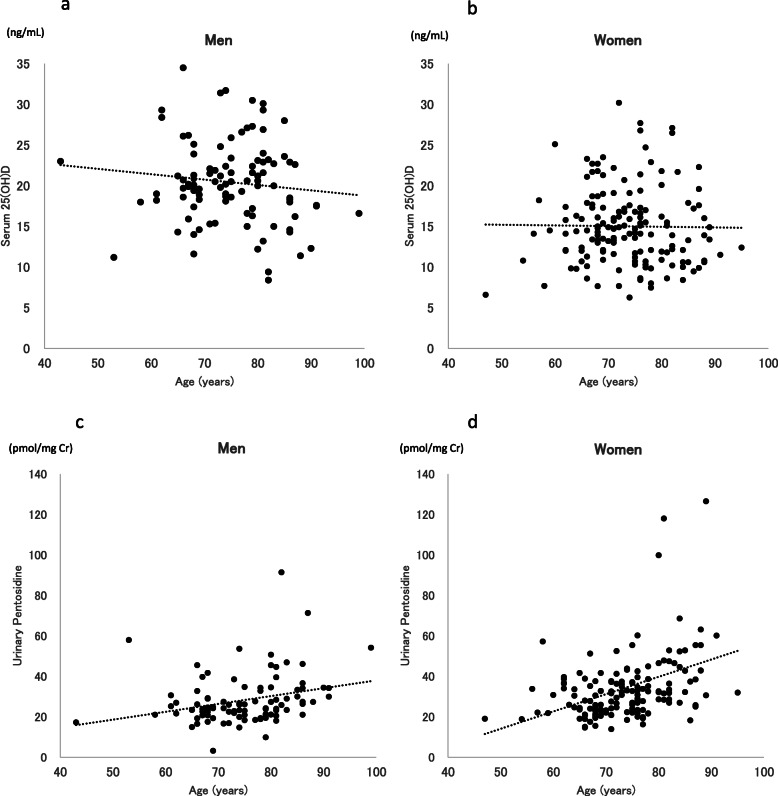
Associations of 25(OH)D and urinary pentosidine with age in both sexes. **a** 25(OH)D is not correlated with age in men (*r* = − 0.116, *p* = 0.258). **b** 25(OH)D is not correlated with age in women (*r* = − 0.014, *p* = 0.864). **c** Urinary pentosidine is positively correlated with age in women (*r* = 0.275, *p* = 0.007). **d** Urinary pentosidine is positively correlated with age in men (*r* = 0.341, *p* < 0.001)

The results of the statistical comparison of serum eGFR, HbA1c, PTH, and 25(OH)D and urinary pentosidine levels between men and women are shown in Table 1. Serum 25(OH)D levels were significantly higher in men than in women (*p* < 0.001), whereas urinary pentosidine levels were significantly higher in women than in men (*p* = 0.010). Serum 25(OH)D and urinary pentosidine levels are shown in scatter plots by age in Fig. 1a-d. 25(OH)D was not correlated with age in either men (*r* = − 0.116, *p* = 0.258) or women (*r* = − 0.014, *p* = 0.864). On the other hand, pentosidine was positively correlated with age in both men (*r* = 0.275, *p* = 0.007) and women (*r* = 0.341, *p* < 0.001).

**Table 1:** Serum eGFR, HbA1c, PTH, 25(OH)D, and urinary pentosidine by sex

	Men (*n* = 97)	Women (*n* = 157)	*p*-value^a^
eGFR (mL/min/1.73 m^2^)	70.7 ± 17.1	70.4 ± 14.8	0.894
HbA1c level (%)	5.6 ± 0.4	5.7 ± 0.5	0.482
PTH level (ng/mL)	43.3 ± 14.5	44.8 ± 12.9	0.613
25(OH)D level (ng/mL)	20.4 ± 5.1	15.0 ± 4.7	< 0.001
Pentosidine level (pmol/mgCr)	28.2 ± 12.6	34.3 ± 20.5	0.010

*eGFR* Estimated glomerular filtration rate, *HbA1c* Hemoglobin A1c, *PTH* Parathyroid hormone, *25(OH)D* 25-hydroxyvitamin D

All values are presented as means ± standard deviations

^a^Difference between men and women (student’s t-test)

Pearson’s correlation coefficients are shown in Table 2. The 25(OH)D level was shown to have a very weak positive correlation with IGF-1 in women. However, in men, it was shown to have a weak positive correlation with height and grip strength and a weak negative correlation with PTH. The pentosidine level had a weak, negative correlation with height, grip strength, gait speed, eGFR, and IGF-1 in men and a weak, negative correlation with height, SOS, grip strength, and gait speed in women. The results of the multiple regression analysis are shown in Table 3. Grip strength was associated with age, height, and pentosidine in men and height and pentosidine in women. Gait speed was associated with age, BMI, and pentosidine in men and age, height, and pentosidine in women.

**Table 2:** Pearson correlation coefficient

	Age	Height	Weight	BMI	SOS	SMI	Grip strength	Gait speed	eGFR	HbA1c	IGF-1	PTH
*Men*												
25(OH)D	− 0.116	0.317**	0.130	−0.063	0.088	0.030	0.307**	0.196	−0.017	−0.070	0.078	−0.246**
Pentosidine	0.275**	−0.322**	−0.188	−0.004	−0.192	−0.093	−0.363**	−0.364**	−0.340**	0.323	−0.247*	0.022
*Women*												
25(OH)D	−0.014	−0.024	−0.008	0.004	0.080	−0.035	−0.082	0.017	−0.093	−0.021	0.211*	−0.289
Pentosidine	0.341**	−0.199*	−0.127	−0.021	−0.209**	0.040	−0.305**	−0.387**	−0.098	0.707	−0.109	0.071

*BMI* Body mass index, *SOS* Speed of sound, *SMI* Skeletal muscle mass index, *eGFR* Estimated glomerular filtration rate, *HbA1c* Hemoglobin A1c, *IGF-1* Insulin-like growth factor-1, *PTH* Parathyroid hormone, *25(OH)D* 25-hydroxyvitamin D

^*^p < 0.05, ^**^p < 0.01

**Table 3:** Results of multiple regression analysis

	B	SE (B)	β	t	95% CI	*p*-value	R^2^
*(a) Grip strength as a dependent variable in men*							
Age	−0.297	0.076	−0.360	−3.909	(−0.447 to − 0.146)	< 0.001	0.420
Height	0.370	0.110	0.317	3.364	(0.151–0.589)	0.001	
Pentosidine	−0.103	0.050	−0.179	−2.051	(−0.203 to − 0.003)	0.043	
*(b) Grip strength as a dependent variable in women*							
Height	0.403	0.042	0.598	9.494	(0.319–0.487)	< 0.001	0.446
Pentosidine	−0.057	0.016	− 0.215	−3.429	(−0.089 to − 0.024)	0.001	
*(c) Gait speed as a dependent variable in men*							
Age	−0.011	0.003	−0.371	−3.765	(−0.016 to − 0.005)	< 0.001	0.253
Pentosidine	−0.005	0.002	−0.254	−2.637	(−0.009 to − 0.001)	0.010	
BMI	−0.026	0.011	−0.233	−2.454	(−0.047 to − 0.005)	0.016	
*(d) Gait speed as a dependent variable in women*							
Age	−0.012	0.003	−0.295	−3.567	(−0.019 to − 0.005)	0.001	0.301
Height	0.013	0.004	0.258	3.316	(0.005–0.021)	0.001	
Pentosidine	−0.004	0.001	−0.182	−2.414	(−0.006 to − 0.001)	0.017	

*B* Partial regression coefficient, *SE* Standard error, *β* Standardized partial regression coefficient, *t* t-ratio, *95% CI* 95% confidence interval, *R2* Coefficient of determination, *eGFR* Estimated glomerular filtration rate, *IGF-1* Insulin-like growth factor-1, *25(OH)D* 25-hydroxyvitamin D

Multiple regression analysis was performed with grip strength and gait speed as dependent variables, and with age (years), height (cm), weight (kg), BMI (kg/m^2^), SOS, SMI, HbA1c, eGFR, 25(OH)D, and pentosidine as independent variables in men and women

The selection of modeling was made using the stepwise method

## Discussion

The purpose of this study was to investigate the association between urinary pentosidine and serum 25(OH)D levels with muscle mass, muscle strength, and physical performance in community-dwelling adults. Serum 25(OH)D levels were significantly higher in men than in women; one of the reasons for this sex difference may be general inactivity and lower intake of vitamin D from daily food among Japanese elderly women compared with men [20].

Urinary pentosidine levels are generally measured by HPLC, but this approach cannot be adapted to analyze many clinical samples, and it is also a time-consuming process. Furthermore, the detection of pentosidine using a reported ELISA kit and an HPLC system requires heat pretreatment, which generates artificial pentosidine, leading to overestimation. A novel pentosidine ELISA system that does not require sample pretreatment for analyzing urine samples has been developed [19]. In this study, the urinary pentosidine level was measured using this new ELISA system.

We found that urinary pentosidine levels were significantly higher in women than in men. There are few studies on the association between urinary pentosidine level and sex in community-dwelling adults; nevertheless, one of the reasons for this difference is that AGEs may be associated with low physical function in elderly women. We found that pentosidine was significantly associated with grip strength and gait speed in men and women. In recent years, the relationship between AGEs and sarcopenia has attracted much attention. However, there has been no report showing the relationship between urinary pentosidine level and specific parameters of physical function.

Independent of age and eGFR [21], the urinary pentosidine level showed a negative association with grip strength and gait speed in men and women. A previous report demonstrated that serum pentosidine level was negatively associated with muscle mass in menopausal women with type 2 diabetes [22]. Another study also reported that elevated levels of serum carboxymethyl-lysine, another representative of AGEs, are associated with decreased walking ability in elderly women [23]. The possible causes for this phenomenon are that the number of AGE receptors in muscle tissue increases with age and that AGEs directly inhibit muscle synthesis [24, 25]. This suggests that pentosidine may be associated not only with bone quality but also with physical function.

Our study is limited by its relatively small sample size, cross-sectional study design, and recruitment of more women than men. The strength of this study is that it is the first to evaluate the association of urinary pentosidine levels with physical function simultaneously. In the future, it will be necessary to conduct longitudinal studies to confirm the findings of this study. These studies may eventually help elucidate the relationship between osteoporosis and sarcopenia.

## Conclusions

In this study, the urinary pentosidine level was significantly associated with grip strength and gait speed. We consider that urinary pentosidine level may serve as a biomarker affecting muscle and bone interactions in clinical practice.

## Data Availability

The datasets generated and analyzed during the current study are not publicly available because of professional discretion, as they were part of patients’ records, but are available as a de-identified datasheet from the corresponding author on reasonable request.

## Abbreviations

BMI: Body mass index
ELISA: Enzyme-linked immunosorbent assay
IGF-1: Insulin-like growth factor-1
SMI: Skeletal muscle mass index
SOS: Speed of sound
eGFR: Estimated glomerular filtration rate
HbA1c: Glycated hemoglobin
PTH: Parathyroid hormone
25(OH)D: 25-hydroxyvitamin D
AGEs: Advanced glycation end products
D3: Cholecalciferol
ECLIA: Electrochemiluminescence immunoassay
HPLC: High-performance liquid chromatography
